# The Association of Community Rates of Internet Use, Depression, and Living Alone in Massachusetts

**DOI:** 10.1093/geroni/igaf122.2716

**Published:** 2025-12-31

**Authors:** Mengshi Liu, Dongfang Hong, Michelle Ward, Qinglin Gao, Elizabeth Dugan

**Affiliations:** University of Massachusetts Boston, Boston, Massachusetts, United States; University of Massachusetts Boston, Boston, Massachusetts, United States; University of Massachusetts Boston, Boston, Massachusetts, United States; University of Massachusetts Boston, Boston, Massachusetts, United States; University of Massachusetts Boston, Boston, Massachusetts, United States

## Abstract

The negative health consequences of social isolation are increasingly recognized, and internet use is often cited as a potential intervention to enhance social connectivity, particularly for at-risk older adults. This study examines the relationship between internet use, depression, and the proportion of older adults living alone across 367 communities in Massachusetts (MA). Data were drawn from the 2025 Massachusetts Healthy Aging Data Reports (healthyagingdatareports.org). Internet use in the past month was derived from BRFSS data (2018–2022), the percentage of older adults living alone was obtained from the American Community Survey (2018–2022), and depression diagnoses were sourced from Centers for Medicare & Medicaid Services data (2020–2021). Across MA, the prevalence of depression ranged from 21.90% to 54.46% (state average: 34.64%), while internet use varied from 40.83% to 88.95% (state average: 70.57%). Regression analyses showed a significant negative association between community rates of internet use and depression among older adults in MA (β = -0.10, p < .000), which remained significant after adjusting for gender distribution and educational attainment. However, when stratified by the proportion of older adults living alone, this relationship was only significant in communities with a higher-than-average proportion of older adults living alone (β = -0.11, p < .001), while no significant association was observed in communities with lower proportions. These findings suggest that internet use may be particularly beneficial in reducing community rates of depression among older adults experiencing greater social isolation, highlighting its potential as a mental health resource for those living alone.

